# *Cells* - An Open Access Journal of Cell Biology

**DOI:** 10.3390/cells1010001

**Published:** 2011-01-03

**Authors:** Shu-Kun Lin

**Affiliations:** MDPI AG, Postfach, CH-4005 Basel, Switzerland; E-Mail: lin@mdpi.com; Website: http://www.mdpi.org/lin/

To expand the open access publishing project of our newly founded company MDPI [1,2]based in Basel, Switzerland, we are in the process of launching new journals. Based on our success in running journals that represent key areas in science and technology, such as *Molecules* [3], *Sensors* [4], *Energies* [5], *Viruses* [6], *Pharmaceuticals* [7], *Cancers* [8] and *Toxins* [9], we are launching a new journal entitled Cells. It is an open access journal combining cell biology, molecular biology and biophysics, toward an understanding of cell structure, function and interactions.

Cells are the basic unit of an organism, just like the molecules in a bulk phase of a substance. As a chemist, I have been interested in the assembly and interaction of molecules to spontaneously form certain structures, for example, unit cells in crystals [10], and I worked hard to offer a new theory to explain the phenomena that “birds of the same feathers flock together” [11,12]. Just yesterday evening,I was watching a TV program with my son about sponges, where the interesting aggregation of dissociated sponge cells was shown [13]. According to standard thermodynamic textbooks, different cells or different molecules aggregate to form a mixture due to the entropy of mixing; this unfortunately does not explain why a large number of the same cells, and a large number of the same molecules, would like to aggregate. The interaction of cells or the collective behavior of aggregated cells to form tissues, organs and organisms will be one of the most important topics to be covered by this journal.

Unlike other open access journals operated by MDPI where there is an Editor-in-Chief for a journal, *Cells* will be managing edited by our in-house editor.

Enjoy publishing with us! 

## References

[B1-cells-01-00001] 1.MDPI in the company name MDPI AG stands for Multidisciplinary Digital Publishing Institute.

[2] MDPI website. www.mdpi.com.

[3] *Molecules* website. http://www.mdpi.com/journal/molecules/.

[4] *Sensors* website. http://www.mdpi.com/journal/sensors/.

[5] *Energies* website. http://www.mdpi.com/journal/energies/.

[6] *Viruses* website. http://www.mdpi.com/journal/viruses/.

[7] *Pharmaceuticals* website. http://www.mdpi.com/journal/pharmaceuticals/.

[8] *Cancers* website. http://www.mdpi.com/journal/cancers/.

[9] *Toxins* website. http://www.mdpi.com/journal/toxins/.

[10] (2011). A new journal *Crystals* is also founded in 2011. http://www.mdpi.com/journal/crystals/.

[11] Lin S.-K. (1996). Correlation of entropy with similarity and symmetry. J. Chem. Inf. Comp. Sci..

[12] Lin S.-K. (2008). Gibbs Paradox and the Concepts of Information, Symmetry, Similarity and Their Relationship. Entropy.

[B13-cells-01-00001] 13.A quick search on *Google Scholar* found many hits on self-aggregation of sponge cells

